# Efficacy of a Novel, Self-Paced, Online Global Health Track Curriculum for Medical Residents

**DOI:** 10.7759/cureus.86810

**Published:** 2025-06-26

**Authors:** Stephanie R Ross, Ana Carolina G Sale, Terry O Derias, Alberto Cruz, Kenneth W Goodman

**Affiliations:** 1 Internal Medicine: Pediatrics, University of California San Francisco, San Francisco, USA; 2 Miller School of Medicine, University of Miami, Miami, USA; 3 Department of Public Health Sciences, Division of Biostatistics, University of Miami Miller School of Medicine, Miami, USA; 4 Institute for Bioethics and Health Policy, University of Miami Miller School of Medicine, Miami, USA

**Keywords:** cultural humility, ethics training, global health education, global health trainee, medical resident education

## Abstract

Introduction

Global health education has become ubiquitous in medical training, yet many residency programs lack structured curricula. We developed a novel, self-paced, online global health curriculum designed for medical residents with limited educational support. The curriculum includes four modules covering global health theory, history, ethics, and cultural humility, supplemented by optional in-person sessions.

Methods

This study evaluates the effectiveness of our global health curriculum designed for medical residents at the University of Miami/Jackson Memorial Hospital Global Health Track. Pre- and post-tests, along with self-efficacy surveys, were used to assess the participants' knowledge and confidence.

Results

Twelve residents participated in piloting the curriculum, with the majority born outside the U.S. and over 70% having prior global health experience. Results demonstrated that there was a positive change in most pre- and post-test scores and narrower interquartile ranges for the self-efficacy scores after completing the course. Participants reported improved confidence in meeting the curriculum's objectives, and the majority stated that the modules were worth their time.

Discussion

The study was limited by several factors, including a small sample size and a single-site study. The self-paced, online format of the curriculum allows for flexibility and accessibility, particularly for institutions with limited funding or faculty support. This curriculum highlights an alternative option for global health training, and the course serves as a scalable model for other institutions seeking to provide global health training. Future directions for this type of curriculum include expansion to other residency programs and inclusion of other learners such as medical students or practicing health professionals.

## Introduction

Despite the evolving interest in global health, the standardization, accessibility, and implementation of global health training have not advanced at the same rate. Much of global health training spans pre-med experiences to medical specialization training, making medical school and residency an ideal time to provide comprehensive education on the history, ethics, and practice of global health [1-7]. One continually ubiquitous aspect of global health interest is participation in a global health rotation, typically abroad. Of the 145 U.S.-based medical schools, 96% offered international electives, and, when polled, 44% of medical students stated that global health opportunities would affect their residency selection [1,2,5]. However, a recent review highlighted that only about 53% of medical schools offer any structured global health education, and only 39% require this before rotating abroad [4]. Although there is no official data from the Accreditation Council for Graduate Medical Education (ACGME), prior surveys of U.S. training programs have shown that about 57% of internal medicine and 58% of pediatric residency programs offer global health training, with 24% of pediatric programs offering global health tracks [8,9]. Even when global health tracks are offered, they are often lacking in meeting the minimum requirements for adequate support and pre-departure training [7,9,10]. This is especially important when it comes to pre-departure training for international rotations in order to prevent problematic behaviors and a lack of preparedness from being exhibited by trainees when abroad.

There is overwhelming evidence to support adequate, accessible global health education, with current offerings ranging from guidelines for global health tracks, criteria for competencies, paid didactic courses, and university-specific curricula [11-17]. Though many medical organizations have articulated the need for structured, supported global health education, there remains a piecemeal approach to how this is achieved, with the implementation of these guidelines left up to individual institutions [18-24]. This need is further emphasized by the recognition and call for action in decolonizing the current global health models of the global north [25-29]. Many of the global health educational offerings for medical residencies are limited to residents at a specific academic institution, charge a fee for access and/or a certificate of completion, are not available fully online, require faculty for facilitation, are only available at certain times of the academic year, or are limited to one specific topic in global health theory (e.g., ethics).

We designed and piloted a novel, didactic global health track curriculum for medical residents to improve their knowledge of and confidence in key aspects of global health theory, including terminology, history, and ethics. Our curriculum differs from existing global health curricula because it is free, online (with an optional in-person component), easily accessible, comprehensive, and self-paced. The primary goal of our curriculum is to aid medical residents in the University of Miami/Jackson Memorial Hospital Global Health Track (GHT) in understanding the key components of global health practice and to bring awareness to current ethical issues and challenges, allowing them to contribute to discussions and efforts around global health in a more thoughtful, self-aware manner.

## Materials and methods

Our course was designed to be predominantly online and largely self-paced, yet optional in-person sessions were offered to accompany each module. The curriculum was developed by medical residents at the University of Miami’s Miller School of Medicine and reviewed by internal and external faculty mentors with expertise in ethics, medical education, and/or global health. The curriculum was devised through the synthesis of available online educational resources and guidelines published by professional societies and institutions, with the primary goal of educating medical residents about the key components in the theory and context of global health practice today [18-22,30]. It consists of online modules with readings accompanied by a variety of multimedia, including journal articles, videos, podcasts, and online open-source modules. Internal Medicine, Pediatrics, and Med-Peds (Combined Internal Medicine and Pediatrics) residents participated. Global Health Track (GHT) residents were required to complete the course; data collected and used were obtained with their consent and de-identified. The study was approved as exempt by the University of Miami Institutional Review Board.

**Figure 1:**
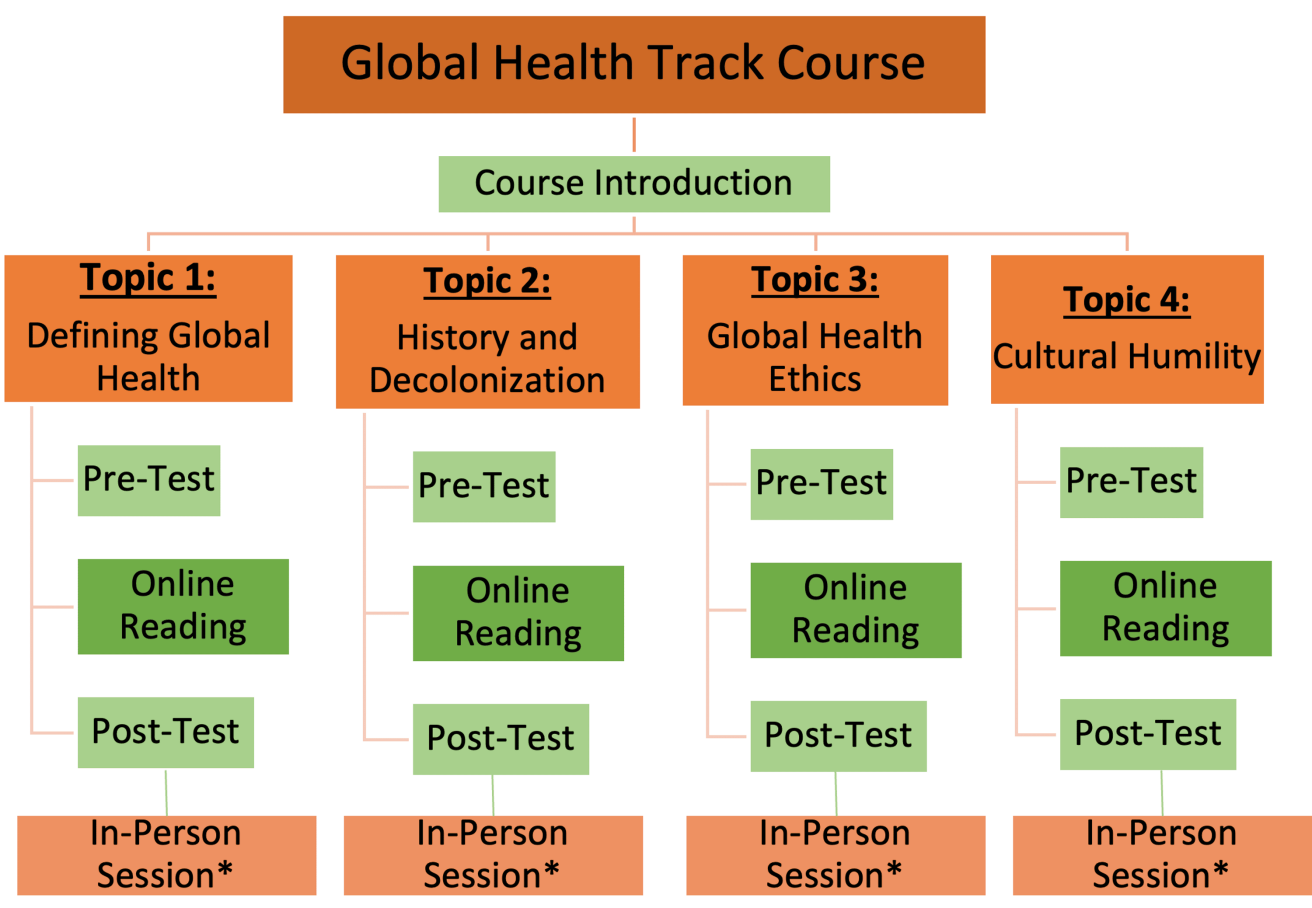
Outline of the global health course structure with four sequential modules *Indicates optional portions of the course

The curriculum is composed of four sequential modules that cover distinct topics in global health and ethics: "Defining Global Health”, “Global Health History and Decolonization”, “Global Health Ethics”, and “Cultural Humility" (Figure 1). Each module has defined objectives that were assessed using online pre- and post-tests. The tests comprise 7-12 questions ranging from multiple-choice and matching to multiple-answer grids and free-text answers.

Our pilot study was conducted over the course of one academic year from August 1, 2022, to June 30, 2023, to assess the efficacy of our novel curriculum. Modules were emailed to residents every two to three months. Protected time was provided for residents to attend the in-person sessions during their regular work hours; required reading and activities were completed beforehand during their extracurricular time. A demographic survey was shared to obtain baseline information. Participants were initially emailed a pre-test before each module, after which they were provided a link to the required module reading (Appendices A-D). Participants then had six to eight weeks to complete the modules before they were emailed that topic’s corresponding post-test (Appendices A-D). The post-tests were scored as a percentile based on the total number correct, ranging from 16 to 24 possible points. Each module was supplemented with an hour-long in-person session with a small group, case-based discussions, which provided an opportunity to debrief on any global health challenges experienced. Study participants also completed self-efficacy surveys to track their confidence in each topic’s sub-objectives on a five-point Likert scale (Appendices E-H). The self-efficacy surveys were completed retrospectively by participants after each module and in-person session.

Study data were collected and managed using REDCap (Research Electronic Data Capture) electronic data capture tools hosted at the University of Miami. REDCap is a secure, web-based software platform designed to support data capture for research studies. We analyzed the baseline demographic survey, pre- and post-test scores, and self-efficacy surveys with descriptive statistics to determine the proportions of the demographic and survey questions. Pre- and post-knowledge test and self-efficacy test scores were analyzed using median scores, median changes, and interquartile ranges (IQR). These data were analyzed using R statistical software, version 4.3.2 (R Core Team (2021). R: A language and environment for statistical computing. R Foundation for Statistical Computing, Vienna, Austria).

## Results

Of the 12 GHT residents, 11 responded to the demographic survey. About half of them were born outside the United States, and more than half of them spoke a language other than English as their native language (Table 1). Nearly three-fourths of GHT residents had participated in a global health rotation, either international or domestic, but only four of them had received any training or formal education in global health before our course. Of the study participants, three had worked for an international nongovernmental organization prior to residency training.

**Table 1:** Participant demographics and background (N = 11)

Characteristic	N (%)
I was born in:	
A country other than the U.S.	6 (55)
The United States	5 (46)
My first language is:	
English	3 (27)
Spanish	7 (64)
Other	1 (9)
I am a(n):	
International Medical Graduate (IMG)	6 (55)
U.S. Medical Graduate	5 (46)
I have participated in an international or domestic global health rotation prior to residency.	
No	3 (27)
Yes	8 (73)
I have received training or formal education in global health study, ethics, or practice prior to residency.	
No	7 (64)
Yes	4 (36)
I have completed or am currently enrolled in a course/degree in tropical medicine.	
No	9 (82)
Yes	2 (18)
I have a master's-level degree in global health, public health, research, or science.	
No	10 (91)
Yes	1 (9)
I have worked for Medicins San Frontieres (AKA Doctors without Borders), the Red Cross, Partners in Health, the Center for Disease Control (CDC), the United Nations (UN), the World Health Organization (WHO), or an international non-governmental organization (NGO) prior to residency.	
No	8 (73)
Yes	3 (27)
I helped participate in the writing, revision, or implementation of the UM/JMH global health track curriculum. (n = 6)	
No	4 (67)
Yes	2 (33)

A range of one to eight participants completed each of the pre- and post-tests and self-efficacy surveys. There was a positive change in the median scores of the pre- and post-tests for modules 1, 2, and 4 (Table 2). The median value of module 3 could not be calculated because there was only one respondent for the Topic 3 post-test. For the self-efficacy surveys, nearly all modules demonstrated a positive change in the median score after completing the course, indicating that participants were more confident in achieving the module’s objectives. The self-efficacy survey results for Topic 3 showed no change in the median score. Overall, the interquartile range of the self-efficacy survey scores also became tighter for all the modules, including Topic 3.

**Table 2:** Pre- and post-test and self-efficacy survey scores across module topics SE = Self-Efficacy; IQR = Interquartile Range. ^a ^The median and IQR for Topic 3 were not calculated due to only having one response for the Topic 3 post-test. The median, change in the median, and interquartile ranges were calculated for both the pre- and post-tests and self-efficacy surveys. The median values trended toward a positive change after the intervention; however, the median change could not be calculated for Topic 3 as the post-test only had one response. The distribution of the average self-efficacy survey scores before and after completing each module (Topics 1-4) was reported via the interquartile range. All the interquartile ranges except for Topic 3 became narrower in the post-self-efficacy surveys when compared to the pre-self-efficacy surveys, indicating greater consistency and less variability between the scores.

Module Topic	Pre-Test	Post-Test	Median Change - Test	Pre-SE	Post-SE	Median Change - SE
	Median (IQR)				Median (IQR)	
Topic 1	15.3 (12.5-16.0)	19.0 (18.5-19.0)	3.8	3.2 (3.1-5.0)	5.0 (4.0-5.0)	1.8
Topic 2	15.0 (12.5-16.0)	15.0 (15.0-16.5)	0	4.5 (2.9-5.0)	4.9 (4.3-5.0)	0.4
Topic 3	15.5 (14.8-16.8)	-^a^	-	4.4 (4.2-4.6)	4.4 (4.3-4.7)	0
Topic 4	9.0 (8.0-11.0)	12.0 (11.5-12.5)	3	4.6 (2.9-4.7)	4.9 (4.0-5.0)	0.3

After completing all the modules and in-person sessions, participants were polled to ask if they thought the sessions were worth their time, and nearly all participants responded "yes." The proportion of the participants who indicated they had read or watched any of the optional material listed in the modules, between 60% and 100% was over the four topics.

When polled about the ease and timing of the in-person session schedule, qualitative feedback ranged from “I wish we had more time to be out during wards but I really enjoy meeting in person and the read(ings)” and “GHT is part of our curriculum… I think it must be included in our learning as residents and not ask for after-hours” to “during the workday is too hectic, already stressful and overbooked.” Another participant noted that it was “difficult for me to keep up with the readings.”

## Discussion

A pilot study of our global health curriculum showed a positive change in meeting the course’s objectives. Both the self-efficacy surveys and pre- and post-tests overall showed that participants' confidence and knowledge improved after completing the course. Additionally, nearly all participants reported that the modules and in-person sessions were worth their time. This is significant, as many residency programs and medical schools offering global health rotations do not provide a pre-departure course [3,4,7-9]. Our course is free, available online, and easily accessible for anyone outside the institution to access; it, therefore, can offer a tool for filling the gap in global health education [12-15]. As the course is self-paced, it allows trainees at institutions that have limited faculty to organize or lead global health courses to have a reliable resource for autodidactic learning. Though we facilitated in-person sessions in an ancillary effort to reinforce knowledge, such sessions can be excluded if there is no local faculty for facilitation. While our results were not statistically significant, they are promising and may be seen to encourage further curricular development and analysis.

The study was limited by a small sample size. Of the 12 Global Health Track residents, not all completed each of the pre-and post-tests and self-efficacy surveys. Our data was confounded by several of the contributing authors of the curriculum, who were also members of the Global Health Track and therefore participants in the study. About a third of participants reported having been involved in the writing or development of our curriculum; however, none of them were involved in the composition or grading of the pre- and post-tests. Lastly, the University of Miami/Jackson Memorial Hospital GHT as well as IM, Peds, and Med-Peds residency programs are not reflective of the average residency demographics in the U.S; more than half of the trainees are originally from and attended medical school outside the United States, which could have confounded or limited the applicability of the data.

The course was also limited by a lack of partner site involvement in the writing and development of our curriculum. To make bona fide strides toward decolonizing our role in global health, it will be important to include partner site members in both the development and conduct of the course [25-28]. Future iterations of the curriculum should include input from host-site counterparts, both domestic and international, in both the revision of the modules and the implementation of the in-person sessions. While we hope that providing an introductory degree of global health education to our medical residents to prevent problematic or stereotypical behaviors on international rotations, it was beyond the scope of our study to determine if our curriculum prepared them to be reflective, considerate, and ethical while rotating abroad. Next steps could include assessing residents' perspectives on the curriculum after completing their global health rotation, in addition to repeating our study with future iterations of residents and possibly external institutions.

## Conclusions

The study of our novel global health course demonstrated the potential for positive change in meeting the course objectives and educating medical residents about the basics of global health. While there were many limitations to our study, it was found to be worthwhile to continue to implement and investigate our curriculum. Future implementation and improvement of our global health course entails including medical students as participants, updating the pre- and post-tests to provide a grade automatically (removing free-text answers), and revising the curriculum to include host-site input. Our course is freely available online with the creation of a University of Miami Moodle account and can be completed by participants at other institutions fully online, self-paced, and readily accessible. The course will be of use to educate residents about the ethics and practice of global health, mitigating their chances of perpetuating colonialist stereotypes when undertaking a global health rotation.

## Appendices

Appendix A: Topic 1 pre/post-test + grading rubric

1. Choose whether the following descriptions most closely describe global health, public health, international health, or tropical medicine. ***1 point for each correct match (total possible: 4)***

A. Healthcare that focuses on providing aid to countries other than one’s own, especially those of LMICs *→ International health*

B. Study and practice of addressing the societal issues that affect health and focuses on prevention rather than a curative intent **→ Public health**

C. Includes any health issue that concerns many countries or is affected by transnational determinants or solutions **→ Global health**

D. Specialty focused on infectious diseases disproportionately seen in LMICs **→ Tropical medicine**

2. **(*MULTIPLE CHOICE) ***Which of the following terms are most preferred today when discussing global health: **1 point for each correct answer chosen and -1 for each incorrect ch****osen, but unable to score less than 0 on this question (total possible: 3)**

A. Poor countries

B. Global South

C. Third world

D. Low and middle-income countries (LMIC) **←answer**

E. Low resource ***←answer***

F. Underserved

G. Developing

H Resource-limited ***←answer***

3. **(***FREE TEXT) *Name at least 5 social determinants of health that affect your patients in Miami.** 1 point for each reasonable example provided (max available: 5)**

Eg. Asbestos exposure - ILD

Eg. Construction job - OA/COPD

Eg. Expensive fruits and veggies - Obesity/DM

Eg. Undocumented - lack of health access -> ESLD/ESRD/CHF

Eg. Neighborhood schools -> Lack of income, unable to see docs or get Rx

4. *(FREE TEXT) *Explain how one of the social determinants of health you listed for question #3 above plays a role in the health of global populations? *2 points for reasonable explanation of a social determinant of health in another non-HIC population (max available: 3)*

5. *(MATCHING) *Please match the following terms with the groups listed below: *Stateless, Internally displaced people (IDP), Refugee, Asylum seeker, Immigrant 1 point for each (max available: 5)*

A. Haitians awaiting a decision for legal protection to the U.S. ***→ Asylum seeker***

B. Rohingya in Myanmar **→ Stateless**

C. Ukrainians in Kharkiv living in underground train stations **→ IDP**

D. Syrians journeying across the Mediterranean Sea to Greece **→ Refugee**

E. South Americans traveling through the Darien Gap with the intention of applying for a visa at the border *-> Immigrant*

6. *(FREE TEXT) *List at least three populations in the United States that are considered to be vulnerable or live in resource-limited settings? *1 point for each (max available: 3)*

A. Immigrants

B. Refugees

C. Asylees

D. Migrants

E. Victims of sex or labor trafficking

F. Native Americans

G. Alaskan Natives

H. People experiencing homelessness

I. Non-native English speakers

7.* (FREE TEXT) *From the perspective of the local partner institutions, what are at least 2 concerns that are seen in global health practice today? *1 point for each (max available: 2)*

*Eg. Tokenism,* *Vertical vs horizontal partnerships,* *Publication bias*

Total points available: 24

Appendix B: Topic 2 pre/post-test + grading rubric

1. Which of the following best define the “White Savior Complex''? Choose all that apply. *Score: 4 points. Only B and D are correct, give 2 points for each answer chosen.*

A. A white American that wants to live amongst people of color in order to “save” them

B. An individual’s perspective that only the talents and resources of people from HIC can help elevate populations in LMIC out of their current situation *<-- answer*

C. Someone who feels called by their religion to help convert and care for people in low-resourced communities

D. The attitude of someone from a more resourced setting who wants to “save” people in LMIC by improving their access to care and resources* <-- answer*

E. An individual from a HIC who wants to live and work for many years in a LMIC

F. All the above

2. In which type of settings did global health work originate? Choose all that apply.* Score: 3 points. Only E is the correct answer, no points if anything else is chosen.*

A. Missionary work

B. European colonies

C. Healthcare for expatriates

D. Military operations

E. Tropical Medicine

F. All the above* <-- answer*

3. Which of the following is an example of neocolonialism in global health today? *Score: 2 points*

A. University of Global Health Equity, a medical and public health school in Rwanda with a focus on training African students to become leaders in global health and to lead health care efforts in their home countries

B. Editorial panel of a global health journal including mostly men and women from European nations** <-- answer**

C. NGO focusing on community health in Nepal has its headquarters and all offices within Nepal and hires primarily Nepali-speaking staff

D. U.S. infectious disease physicians and epidemiologists serving as technical advisers and Ministry of Health leaders as decision makers and organizers in a viral hemorrhagic fever epidemic in East Africa

4. Which of the following is the ultimate goal of decolonization in global health? *Correct answer is C, give 2 points if chosen. Max score: 2.*

A. Dismantle colonialist powers and dictatorships that perpetuate systemic inequities across the world

B. Remove medical mission trips from global health practice and ensure only long-term health systems strengthening focused practices

C. Removal of all forms of supremacy from global health and ensure that the leaders and key players in the research, study, practice, and policy arms of global health are reflective of the populations that are targeted by that work* <-- answer*

D. Ensuring funding streams encourage more community engagement and hiring of local staff

5. *(FREE TEXT)* What remnants of colonialism are still present in global health today?Describe at least 4. *Score: 4 points. Need at least 4 answers, one point for each.*

Examples of possible answers:

Language - English or French are preferred

Drug production mostly done on other continents than where healthcare is provided

Blaming lack of structure or access to healthcare on populations of LMIC or POC in America

Seeing the experts/primary providers as those from HIC

Using clinical rotations abroad for education of those from HIC without compensation

Allowing trainees to practice outside their scope when rotating abroad

International NGOs paying locals significantly less than expats working for the same NGO

Bringing staff from LMIC to low-paying, subservient jobs in the US/Europe

Short-term projects in LMIC without input from locals or goal to transition to local control

Perpetuating a cycle of reliance on international aid/support

6. **(***FREE TEXT) *List at least 3 changes or actions that individuals or institutions can make in order to decolonize global health?* Score: 3 points. Need at least 3 arguments from any of the readings provided.*

Examples of possible answers:

Hire individuals of LMIC

Focus on educating and training those in LMIC

Focus on providing assistance with coordination and advising, not being decision-makers

Provide primary/equal authorship

Collaborate to obtain joint funding

Acknowledge articles/journals in other languages aside from English

Set up offices and staff in LMIC, not just HIC

7. Do you think that global health will survive its decolonization? Why or why not? *Score: 2 points. *

Any argument will be accepted if reasonable.

Total points available: 20

Appendix C: Topic 3 pre/post-test + grading rubric

1. What should be the primary mission of a resident who participates in a global health rotation? *Total score: 2 points*

A. Provision of clinical care

B. Education of residents, PAs, and/or nurses at the global health site

C. Learning about clinical care and health system at the global health site *<-- answer*

D. Providing extra support for research and/or quality improvement

E. Improve the health of a community by introducing proven public health interventions

F. More than one of the above

2. Which of the following is true regarding ethical expectations of trainees rotating abroad? *Total score: 2 points*

A. One of your primary goals should be participating in research that focuses on effects of interventions that are likely to benefit the overall health of a community.

B. You should perform the clinical duties designated as your responsibility by your supervising attending, even if you have yet to perform these duties comfortably at your home institution.

C. You should learn about the site’s history, culture, and health system as part of pre-departure training to reduce any potential adverse effects of your presence at the global health site. **<-- answer**

D. You should emphasize evidence-based medicine practices while on your rotation to share your knowledge with patients and clinical staff at the global health site.

3. In a situation where cultural norms are at odds with your own and you find the local norms concerning, offensive, or potentially harmful, what is the most reasonable action to take? *Total score: 2 points*

A. Discuss the situation with your mentors or local supervisor. **<-- answer**

B. Resign from your position and return home from your global health rotation early if needed.

C. Report the situation to the local authorities or other appropriate staff as you would as your home institution.

D. Continue being respectful and follow the lead of other clinical staff and colleagues involved in the situation at the global health site.

4. Your supervising attending at your global health rotation encourages you to perform research on an intervention that will benefit the catchment area of the hospital in which you’re working. You begin to prepare for this research project. Of the following, which should not be considered while preparing your research project? *Total score: 2 points*

A. Inclusion of local colleagues in authorship with order of authorship based on actual role

B. IRB approval by your home institution and the institution affiliated with your global health site

C. Development of intervention in cooperation with local clinical colleagues and community members

D. Waive informed consent due to primary language of patients being something other than English *<-- answer*

5. What are possible ethical dilemmas that may arise when undertaking an international research project? Select all that apply: *Total score: 4 points, one for each answer correctly selected*

A. Academic journals are typically published in English, leading non-English speakers to rely on their counterparts from mostly HIC for assistance writing and editing the manuscript *<-- answer*

B. Funding is more likely to be given to researchers from HIC than LMIC *<-- answer*

C. The project may benefit the local community more than international researchers, leading to the project being extended

D. LMIC researchers needs may be put below the needs of those from HIC due to inequities in sources of funding and access to publishing opportunities *<-- answer*

E. Researchers from HIC are more likely to be listed as a first author than those from LMIC *<-- answer*

F. Research may increase awareness of healthcare needs and disparities in LMIC

6. Of the following recommendations, which ones are specifically for trainees on international rotations as described in the WEIGHT guidelines (pick all that apply): *Total score: 4 points, one for each correct*

A. Monitor costs and benefits to ensure equity

B. Communicate with local mentors **<-- answer**

C. Establish expectations of the experience *<-- answer*

D. Promote transparency and identifying conflicts of interests

E. Be cognizant of level of training when acquiring new skills *<-- answer*

F. Engage in discussions regarding differing approaches to care *<-- answer*

G. Aspire to long-term partnerships

7. *(MATCHING) *Matching the following actions to the UM/JMH Ethics Guidelines for trainees rotating abroad for global health rotations: *Total score: 6 points, one for each correct*

A. Don’t take photographs with identifying features *-> Respect dignity and privacy*

B. Discard the idea that one can be culturally competent when providing care *-> Practice cultural humility*

C. Research applicable clinical guidelines and resources to be used while abroad *-> Aim to learn*

D. Purchase evacuation insurance *-> Minimize burden*

E. Review possible ethical dilemmas one may encounter while abroad* -> Acknowledge limitations*

F. Research the host country’s language, laws, and customs prior to departure *-> Minimize burden*

8. **(***FREE TEXT) *Describe the meaning of “neo-colonialism” in the context of Operation Smile’s history as discussed in the “Seven Sins of Humanitarian Medicine”? *Total score possible: 2 points*

Possible answer: 

Short term work perpetuates the idea that HIC are able to work temporarily and only assist when it suits them best, leaving behind poor follow up and lack of sustainable infrastructure as these LMIC sites come to rely on this revolving door instead of constructing and training their own healthcare providers/hospitals. Operation Smile performed a limited number of surgeries without involving or training many local providers and then would leave equipment that was unable to be used locally after their stints abroad. They would not organize follow up care post-op or help build a more sustainable, long-term solution to fixing cleft lips/palates abroad.

Total points available: 24

Appendix D: Topic 4 pre/post-test + grading rubric

1. What is structural violence in medicine? Select all that apply. **Total score: 2 points (one for each correct)**

A. Institutional or societal structures that are so ubiquitous and biased that they can seem invisible **<-- answer**

B. The search for the molecular basis of disease to cure diseases that affect certain groups of people more than others

C. The economic, political, legal, religious and cultural structures that prevent individuals or groups of people from reaching their best health *<-- answer*

D. The physical harm caused by one religious group to another based on their discriminatory views towards them

E. All the above

2. Where was the location of the U.S. Public Health Service study that studied the natural course of syphilis in black men, denying them access to widely available penicillin treatment until multiple new organizations reported their unethical practices? *Total score: 1 point*

A. Blackfoot Reservation, Montana

B. Tuskegee, Alabama* <--answer*

C. Dallas, Texas

D. Memphis, Tennessee

E. Chinle, Arizona

3. What is the name for the term to describe housing segregation against black house owners due to racist banking practices? *Total score: 1 point*

A. Blacklining

B. Neighborhood Redistricting Act

C. Gentrification

D. Redlining *<-- answer*

4. **(FREE TEXT) **Describe at least one implicit bias that you have. *Total score: 2 points*

Answers need to have: 

- at least 1 bias listed from the following options (but not limited to these): gender, sexuality, religion, skin tone, ethnicity, disability, native american, arab, weight, age, asian, transgender

- an explanation as to what they are biased against (and preferably why they think they have it but not required).

5. True/False: Cultural competence is preferred to cultural humility. *False*

Total score: 1 point

6. True/False: Home remedies that are foul-smelling or involve waste are typically harmful for health. *True*

Total score: 1 point

7. True/False: It is important to tell local communities immediately and directly that they are wrong when their health practices are harmful. *False*

Total score: 1 point

8. What is NOT a home remedy that can confer some benefits in treating the illness? *Total score: 1 point*

a.     Honey on wounds

b.     Amniotic sac on ulcers

c.     Fungus tea for childhood fevers

d.     Dirt on cut umbilical cord *<-- answer*

9. What are the 3 tenets of cultural humility (from the UMN modules)? **Total score: 3 points (one for each correct question)**

A. Acknowledging and challenging power imbalances *<-- answer*

B. Lifelong commitment to the study of other cultures

C. Identifying cultural differences

D. Creating non-paternalistic collaborative relationships *<-- answer*

E. A lifelong commitment to self critique** <-- answer**

F. Encouraging one culture to rely on another

Answer the following questions regarding working with an interpreter:

10. Who should the provider speak directly to? *Total score: 1 point*

A. Fluent medical staff

B. Patient* <-- answer*

C. Interpreter

D. Fluent family member

11. What types of language should a provider avoid using with an interpreter? Select all that apply. *Total score: 1 point*

A. Colloquialisms

B. Abbreviations

C. Complicated sentences

D. Fast speech

E. All the above* <-- answer*

12. How many words or sentences should a provider speak before waiting for the interpretation? *Total score: 1 point*

A. No more than 8-10 words

B. 1 sentence max

C. 1-2 sentences *<-- answer*

D. 3-4 sentences

E. As long as they need to communicate their thoughts

Total points available: 16

Appendix E: Topic 1 self-efficacy survey

For each of the objectives listed below, please choose the number that indicates your level of confidence both before and after completing the online and in-person session for Topic 1 (If you just completed the online portion, rate your confidence for only objectives 1-5).

1 = Strongly Disagree

2 = Disagree

3 = Neutral

4 = Agree

5 = Strongly Agree

**Table 3:** Topic 1 self-efficacy survey *Only answer if attended the in-person session for Topic 1.

	Confidence BEFORE completing Topic 1					Confidence AFTER completing Topic 1				
	1	2	3	4	5	1	2	3	4	5
I feel confident differentiating between global health, tropical medicine, and international health.										
I feel confident identifying between preferred terminology and antiquated labels for discussing global health.										
I feel confident differentiating between at least five of the target populations which would benefit from a global health-minded approach to providing care.										
I feel confident discussing how several social determinants of health affect the health outcomes of various global populations.										
I feel confident explaining how global health care can be practiced locally in one’s home country and community.										
I feel confident describing at least two concerns raised by educators in low and middle-income countries (LMIC) in regards to the role of individuals participating in global health projects abroad										
I feel confident analyzing how various local populations should be cared for with a global health-minded approach.*										
I feel confident critiquing my own attitude, skills, and expectations for my global health rotation.*										

Appendix F: Topic 2 self-efficacy survey

For each of the objectives listed below, please choose the number that indicates your level of confidence both before and after completing the online and in-person session for Topic 2 (If you just completed the online portion, rate your confidence for only objectives 1-4).

1 = Strongly Disagree

2 = Disagree

3 = Neutral

4 = Agree

5 = Strongly Agree

**Table 4:** Topic 2 self-efficacy survey *Assessed primarily during the in-person session

	Confidence BEFORE completing Topic 2					Confidence AFTER completing Topic 2				
	1	2	3	4	5	1	2	3	4	5
I feel confident defining neocolonialism and the white savior complex.										
I feel confident explaining the origin of global health as it relates to colonialism, racism, and discrimination.										
I feel confident distinguishing the ultimate goal of the decolonization of global health.										
I feel confident defending the importance of the decolonization movement within global health.										
I feel confident formulating examples of problematic behaviors in the global health sphere.*										
I feel confident reflecting on best practices for decolonizing global health in international work and rotations.*										
I feel confident analyzing the ways in which the privileged, colonialist mindset is represented locally within our local healthcare system.*										

Appendix G: Topic 3 self-efficacy survey

For each of the objectives listed below, please choose the number that indicates your level of confidence both before and after completing the online and in-person session for Topic 3 (If you just completed the online portion, rate your confidence for only objectives 1-5).

1 = Strongly Disagree

2 = Disagree

3 = Neutral

4 = Agree

5 = Strongly Agree

**Table 5:** Topic 3 self-efficacy survey *Assessed primarily in the in-person session

	Confidence BEFORE completing Topic 2					Confidence AFTER completing Topic 2				
	1	2	3	4	5	1	2	3	4	5
I feel confident analyzing ways in which a physician may negatively impact a local community when volunteering or working abroad.										
I feel confident describing the five ethical expectations for our own trainees when participating in a global health rotation.										
I feel confident explaining the importance of stressing a trainee’s primary role as that of a learner when rotating abroad.										
I feel confident disproving the following statement: Doing something is better than nothing.										
I feel confident discussing three ethical challenges which may present when participating in an international research project.										
I feel confident hypothesizing possible ethical issues trainees may face during a rotation abroad.*										
I feel confident demonstrating appropriate self-reflection and reasoning when working through case-based ethical dilemmas that may be encountered on an international rotation.*										

Appendix H: Topic 4 self-efficacy survey

For each of the objectives listed below, please choose the number that indicates your level of confidence both before and after completing the online and in-person session for Topic 4 (If you just completed the online portion, rate your confidence for only objectives 1-5).

1 = Strongly Disagree

2 = Disagree

3 = Neutral

4 = Agree

5 = Strongly Agree

**Table 6:** Topic 4 self-efficacy survey *Assessed primarily in the in-person session

	Confidence BEFORE completing Topic 2					Confidence AFTER completing Topic 2				
	1	2	3	4	5	1	2	3	4	5
I feel confident explaining at least one of my own implicit biases										
I feel confident illustrating one way in which Black Americans were isolated from economic opportunities in the mid-1900’s.										
I feel confident critiquing the role of “structural violence” in clinical medicine.										
I feel confident comparing and contrasting cultural humility and cultural competence.										
I feel confident discussing the role of spiritual and non-allopathic medicinal practices in community health.										
I feel confident producing at least two examples of possible adverse outcomes for patient care when professional interpreters are not used by providers.										
I feel confident distinguishing best practices for working with medical interpreters in a clinical setting.										

## References

[1] (2024). Association of American Medical Colleges. International Electives. https://www.aamc.org/search?keys=international%20electives.

[2] Barzansky B, Etzel SI (2017). Medical schools in the United States, 2016-2017. JAMA.

[3] Drain PK, Holmes KK, Skeff KM, Hall TL, Gardner P (2009). Global health training and international clinical rotations during residency: current status, needs, and opportunities. Acad Med.

[4] Edwards MK, An C, Rohrbaugh R, Peluso MJ, Lam SK (2022). A web-based review of global health programs in U.S. allopathic and osteopathic medical schools. Med Teach.

[5] Lu PM, Park EE, Rabin TL, Schwartz JI, Shearer LS, Siegler EL, Peck RN (2018). Impact of global health electives on US medical residents: a systematic review. Ann Glob Health.

[6] Khan OA, Guerrant R, Sanders J (2013). Global health education in U.S. medical schools. BMC Med Educ.

[7] Peluso MJ, Forrestel AK, Hafler JP, Rohrbaugh RM (2013). Structured global health programs in U.S. medical schools. A web-based review of certificates, tracks, and concentrations. Acad Med.

[8] Hau DK, Smart LR, DiPace JI, Peck RN (2017). Global health training among U.S. residency specialties: a systematic literature review. Med Educ Online.

[9] Watts J, Russ C, St Clair NE, Uwemedimo OT (2018). Landscape analysis of global health tracks in United States pediatric residencies: moving toward standards. Acad Pediatr.

[10] Haq H, Barnes A, Batra M (2019). Defining global health tracks for pediatric residencies. Pediatrics.

[11] Webber S, Lauden SM, Fischer PR, Beyerlein L, Schubert C (2020). Predeparture activities curricular kit (PACK) for wellness: a model for supporting resident well-being during global child health experiences. Acad Pediatr.

[12] Sawaya RD, Mrad S, Rajha E, Saleh R, Rice J (2021). Simulation-based curriculum development: lessons learnt in Global Health education. BMC Med Educ.

[13] Fallah PN, Ovsak GG, Kasper J (2020). A longitudinal case-based global health curriculum for the medical student clerkship year. MedEdPORTAL.

[14] Pitt MB, Slusher TM, Gladding SP, Moskalewicz R, Howard CR (2020). Predeparture activities curricular kit (PACK) for wellness: a model for supporting resident well-being during global child health experiences. Am J Trop Med Hyg.

[15] Hughey KL, Bell JD, Mullan PB (2019). Scaling up a global health and disparities path of excellence pilot program at the University of Michigan Medical School. Acad Med.

[16] Barry M, Sugarman J, DeCamp M, Richardson G, Rodriguez J (2024). Ethical challenges in short-term global health training. Stanford Center for Innovation in Global Health. Ethical Challenges in Short-term Global Health Training.

[17] St Clair N, Pitt M, Butteris S (2019). Introducing S-Pack (SUGAR’s pre-departure activities curricular kit): a comprehensive preparation curriculum for global health experiences. Pediatrics.

[18] DeCamp M, Lehmann LS, Jaeel P, Horwitch C (2018). Ethical obligations regarding short-term global health clinical experiences: an American College of Physicians position paper. Ann Intern Med.

[19] St Clair NE, Abdul-Mumin A, Banker SL (2020). Global guide: a comprehensive global health education resource for pediatric program directors. Pediatrics.

[20] Consortium of Universities for Global Health (CUGH) Competency Sub-Committee (2024). Consortium of Universities for Global Health (CUGH) Competency Sub-Committee. CUGH Global Health Education Competencies Tool Kit (3rd ed). Washington, DC: Consortium of Universities for Global Health. UGH Global Health Education Competencies Tool Kit (3rd ed).

[21] Crump JA, Sugarman J (2010). Ethics and best practice guidelines for training experiences in global health. Am J Trop Med Hyg.

[22] (2024). Recommended curriculum guidelines for family medicine residents: global health. https://www.aafp.org/dam/AAFP/documents/medical_education_residency/program_directors/Reprint287_Global.pdf.

[23] Lasker JN, Aldrink M, Balasubramaniam R (2018). Guidelines for responsible short-term global health activities: developing common principles. Global Health.

[24] Sayeed S, Taylor L (2020). Institutionalising global health: a call for ethical reflection. BMJ Glob Health.

[25] Garba DL, Stankey MC, Jayaram A, Hedt-Gauthier BL (2021). How do we decolonize global health in medical education?. Ann Glob Health.

[26] Khan M, Abimbola S, Aloudat T, Capobianco E, Hawkes S, Rahman-Shepherd A (2021). Decolonising global health in 2021: a roadmap to move from rhetoric to reform. BMJ Glob Health.

[27] Kwete X, Tang K, Chen L (2022). Decolonizing global health: what should be the target of this movement and where does it lead us?. Glob Health Res Policy.

[28] Eichbaum QG, Adams LV, Evert J, Ho MJ, Semali IA, van Schalkwyk SC (2021). Decolonizing global health education: rethinking institutional partnerships and approaches. Acad Med.

[29] Atkins S, Banerjee AT, Bachynski K (2021). Using the COVID-19 pandemic to reimagine global health teaching in high-income countries. BMJ Glob Health.

[30] Ross SR, Goodman KW (2023). Avoiding unethical altruism in global health: revisiting ethics guidelines for international rotations for medical residents. J Grad Med Educ.

